# Hydrogen sulfide inhibits epithelial-mesenchymal transition in peritoneal mesothelial cells

**DOI:** 10.1038/s41598-018-21807-x

**Published:** 2018-04-12

**Authors:** Shengnan Cheng, Ying Lu, Yuanyuan Li, Luyan Gao, Huaying Shen, Kai Song

**Affiliations:** Department of Nephrology, Second Affiliated Hospital of Soochow University, Suzhou, P.R. China

## Abstract

Peritoneal fibrosis (PS) determines the long-term outcome of peritoneal dialysis (PD). We previous confirmed that hydrogen sulfide (H_2_S) inhibited PS, but its cellular mechanism was not fully elucidated. Epithelial-mesenchymal transition (EMT) of mesothelial cells (MCs) is an important cellular event of PS, we therefore investigated whether EMT can be affected by H_2_S in MCs. Rats were treated with 4.25% -glucose PD fluids plus lipopolysaccharide for 28 days to produce PS, and NaHS (56 μg/kg.d) was given simultaneously. NaHS (56 μg/kg.d) reduced the deposition of collagen in the submesothelial zone compared with the PS group. In primarily cultured rat MCs, 4.25% -glucose PD fluid induced EMT in MCs featured as loss of ZO-1 and Cytokeratin, and increase of α-SMA, plasminogen activator inhibitor 1, fibronectin and TGF-β1 proteins. PD fluid also increased IL-6 and monocyte chemotactic protein-1 mRNA expressions as well as the phosphorylation of Smad2/3 and Smad3. NaHS (50–300 μmol/L) reversed the above alterations with the optimal dose at 100 μmol/L. Thus, exogenous H_2_S improves PS by inhibiting EMT in MCs. The anti-EMT effect of H_2_S is associated with the inhibition of inflammation and TGF-β1-Smad signal pathway.

## Introduction

Peritoneal fibrosis induced by the chronic stimulation of high glucose peritoneal dialysis fluid and frequent peritonitis is a major cause of ultrafiltration failure of peritoneal dialysis (PD)^1^. The pathological characteristic of peritoneal fibrosis consists of the loss of mesothelial cells (MCs), neovascularization, thickened submesothelial zone and the presence of myofibroblasts^2^. Interventions against these histological features are believed to ameliorate peritoneal fibrosis and improve the long term outcome of PD patients. However, effective treatments of peritoneal fibrosis are still limited.

Hydrogen sulfide (H_2_S) is the third endogenous gasotransmitter compared to carbon monoxide and nitric oxide^3^. The decreased plasma level of H_2_S in various fibrosis diseases provides the rationale of supplement of H_2_S in treating organ fibrosis^4^. Our previous work has confirmed that NaHS, a H_2_S donor inhibited the deposition of collagen fibers, inflammation and angiogenesis in the peritoneum of a chronic peritonitis rat model^5^, but the cellular mechanisms of H_2_S on peritoneal fibrosis has not been fully understood.

For the last twenty years, epithelial-mesenchymal transition (EMT) of peritoneal mesothelial cells has been used to explain the loss of MCs and the occurrence of myofibroblasts during peritoneal fibrosis^6^. Conventional PD fluids can stimulate the MCs to undergo EMT characterized by the disassembly of cellular tight junctions, increase of mesenchymal markers and the ability of invasion. As H_2_S is able to ameliorate peritoneal fibrosis, we hypothesize that H_2_S can inhibit EMT of MCs during peritoneal fibrosis. In this study, we examined the effect of H_2_S on EMT induced by 4.25% peritoneal dialysis fluid in primarily cultured rat MCs. The potential mechanisms of the anti-EMT effect of H_2_S were also explored.

## Results

### H2S reduced peritoneal fibrosis induced by chronic peritonitis in rats receiving PD

**Figure 1:**
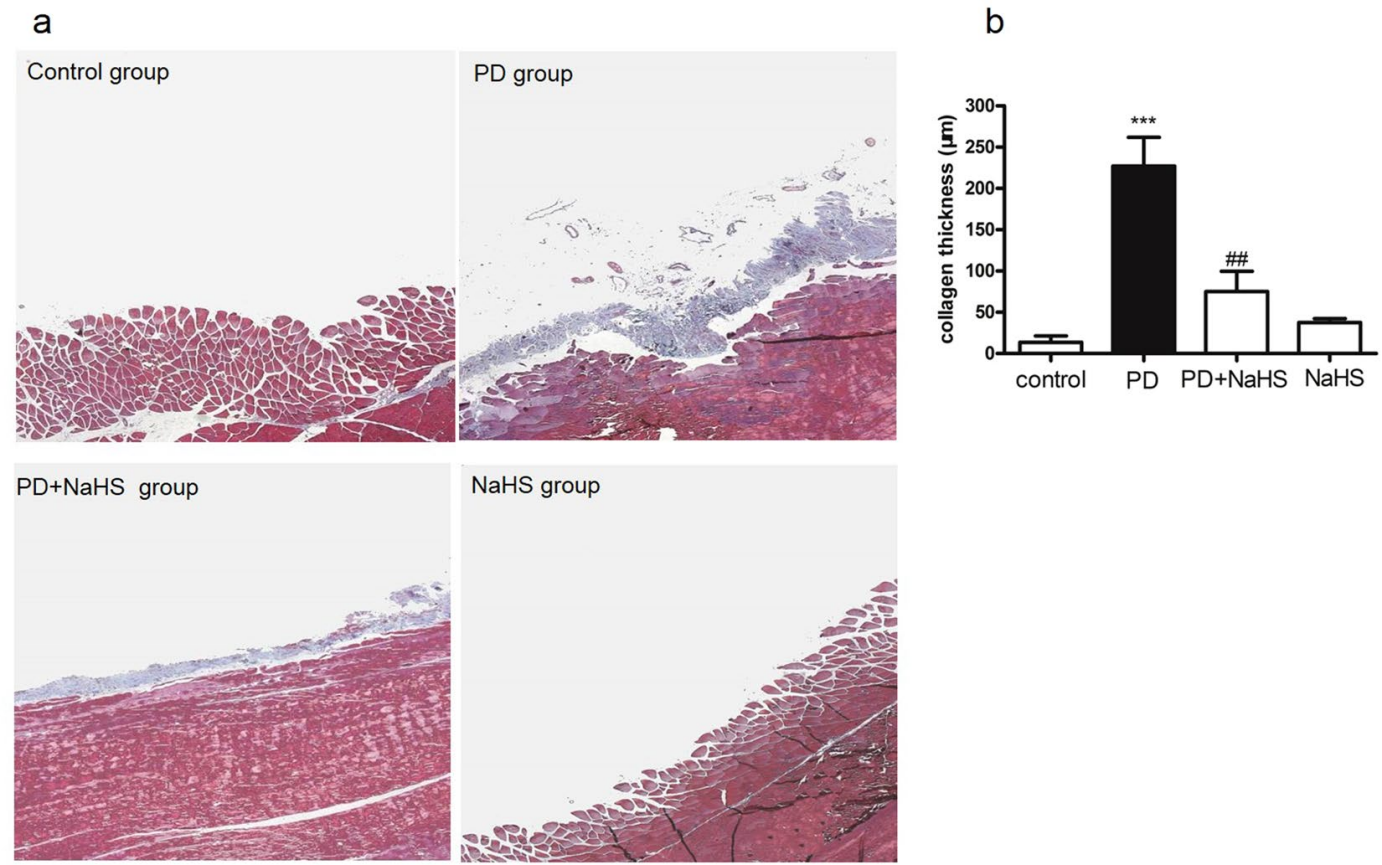
Masson-trichrome staining of rat peritoneal tissue. (**a**) On the 28th day, the fibrotic thickness of the rat peritoneum in the PD group was significantly increased compared with the control group. Administration of NaHS (56 µg/kg/day) in the PD rats markedly reduced the deposition of collagen fibers. The thickness of collagen fibers (blue) of the NaHS group and the control group was comparable. Magnification × 200. (**b**) Quantitative analysis of the collagen thickness (µm) of the peritoneum. Data are presented as mean ± SD, *n* = 5. ***P < 0.001 versus control group; ^##^*P* < 0.01 versus PD group.

Masson-trichrome staining was used to assess the area of peritoneal fibrosis. Compared with the control group, injection with 4.25% peritoneal dialysate plus LPS considerably increased the amount of collagen (blue area) in the rats. Administration of NaHS (56 μg/kg.d) reduced the thickness of collagen fibers in the PD rats (Fig. 1).

### Identification of primarily cultured rat peritoneal mesothelial cells

**Figure 2:**
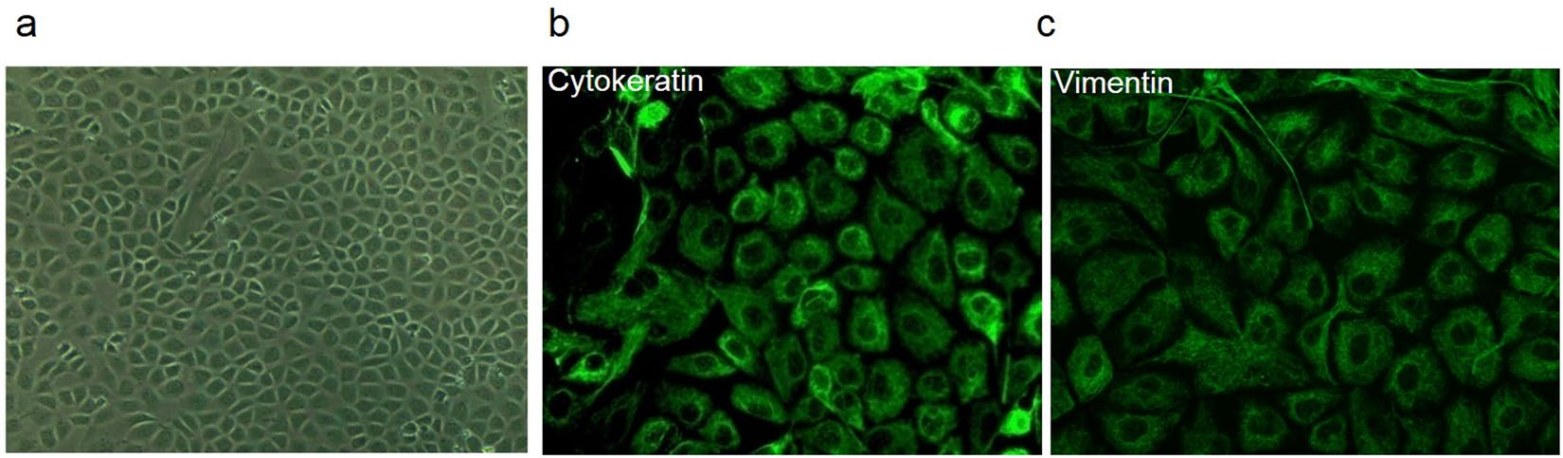
Identification of rat peritoneal mesothelial cells (RPMCs). (**a**) Primarily cultured peritoneal mesothelial cells exhibited cobble-like appearance under light microscope. (**b**,**c**) Cellular immunofluorescence staining revealed that cytokeratin and vimentin were positive in primarily cultured RPMCs. Original magnification × 400.

Primarily cultured cells of the third passage exhibited a polygonal cobblestone-like appearance. The cells expressed both epithelial marker (cytokeratin) and mesenchymal marker (vimentin) (green color) indicating that these cells were peritoneal mesothelial cells (Fig. 2).

### H_2_S alleviates the loss of epithelial markers induced by 4.25% peritoneal dialysate in peritoneal mesothelial cells

**Figure 3:**
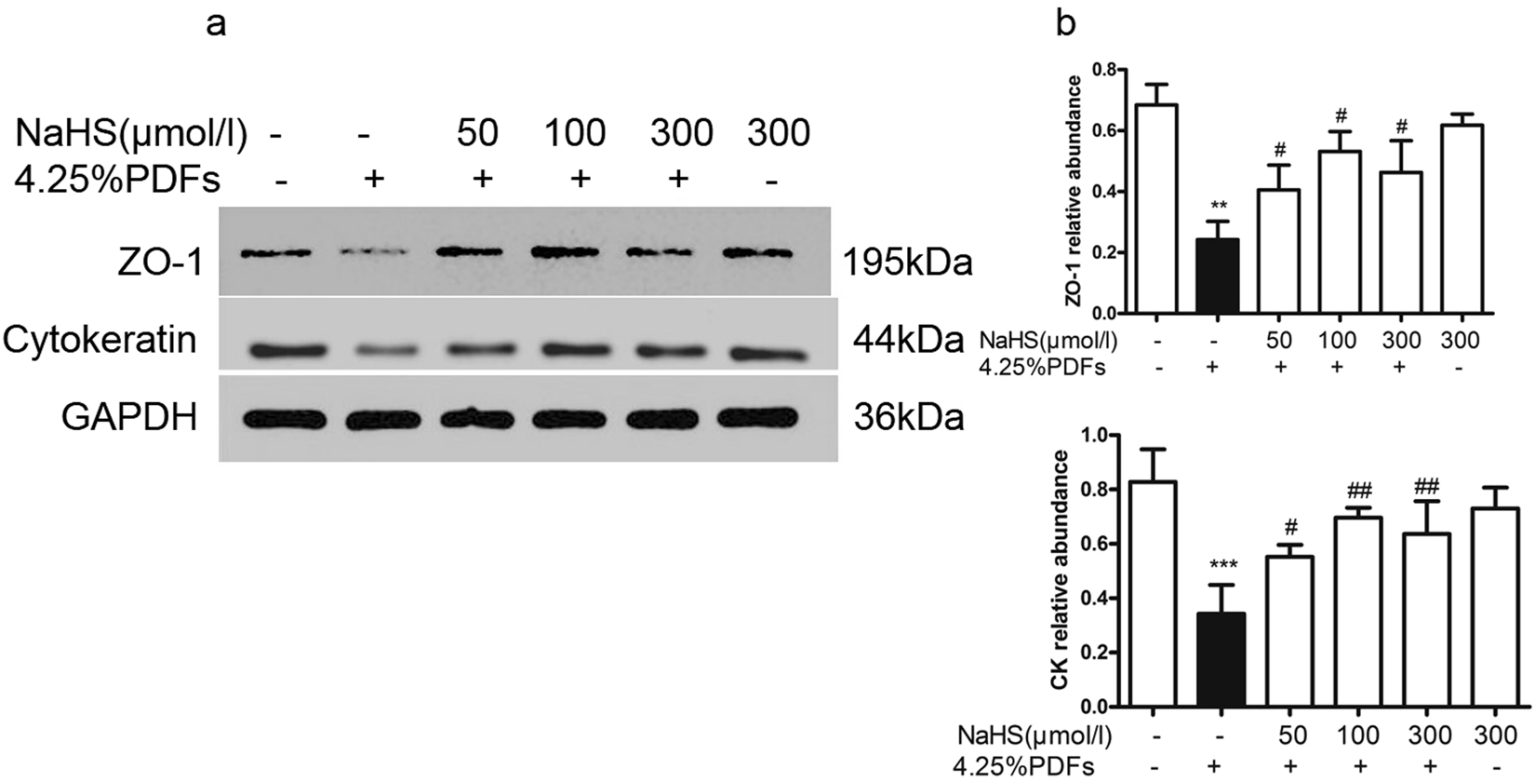
Effect of H_2_S on ZO-1 and Cytokeratin induced by4.25% glucose PDFs in RPMCs. After pretreatment with or without NaHS (50, 100, 300 μmol/L) for 30 mins, RPMCs were exposed to the mixture of 4.25% glucose PDFs and culture medium by 1:1 for 24 h. (**a**) ZO-1 and Cytokeratin protein were detected by Western blot and (**b**) the relative abundance of these proteins in each group are presented. Data represent mean ± SD of three independent experiments. **P < 0.01, ***P < 0.001 versus control group; ^#^*P* < 0.05, ^##^*P* < 0.01 versus 4.25% glucose PDF group.

Primarily cultured peritoneal mesothelial cells (RPMC) were pretreated with various concentrations of NaHS (50–300 μmol/L) for 30 mins, followed by incubation with 4.25% glucose peritoneal dialysate solutions (PDFs) for 24 hours. Compared with the control group, 4.25% PDFs significantly decreased the expressions of ZO-1 and cytokeratin in RMPC. Incubation with NaHS (50–300 μmol/L) reversed the downregulation of ZO-1 and cytokeratin induced by the 4.25% PDFs, with the optimal effect in the 100 μmol/L NaHS group (Fig. 3).

### H_2_S decreases mesenchymal markers and profibrotic factors activated by 4.25% PDFs in RPMC

**Figure 4:**
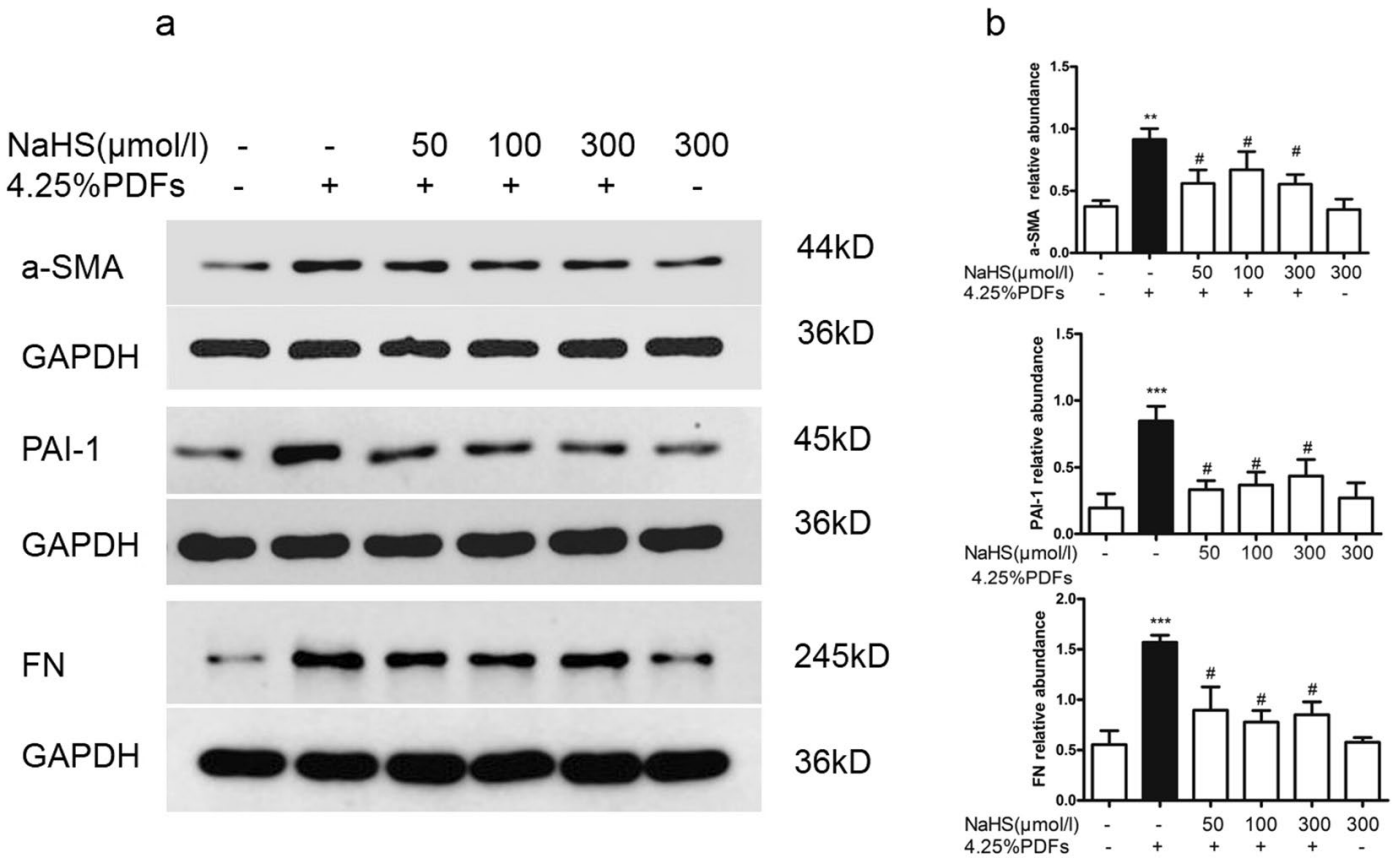
Effect of H_2_S on fibrogenic factors induced by 4.25% glucose PDFs in RPMCs. RPMCs were pretreated with or without NaHS (50, 100, 300 μmol/L) for 30 mins, then exposed to the mixture of 4.25% glucose PDFs and culture medium by 1:1 for 24 h. (**a**) a-SMA, PAI-1 and fibronectin protein were detected by Western blot and (**b**) relative abundance of these proteins in each group are presented. Data represent mean ± SD of three independent experiments. **P < 0.01 versus control group; ^#^*P* < 0.05 vs. 4.25% glucose PDF group.

After treatment with NaHS (50–300 μmol/L) for 30 mins, RPMC were exposed to 4.25% glucose PDFs for 24 hours. Incubation with 4.25% PDFs increased the expressions of mesenchymal markers including α-SMA and profibrotic factors including PAI-1, fibronectin (FN) and TGF-β1, which were decreased by pretreatment with NaHS, especially in the 100 μmol/L NaHS group (Fig. 4).

### H_2_S suppress the TGF-β1 signal pathway activated by 4.25%PDFs in RPMC

**Figure 5:**
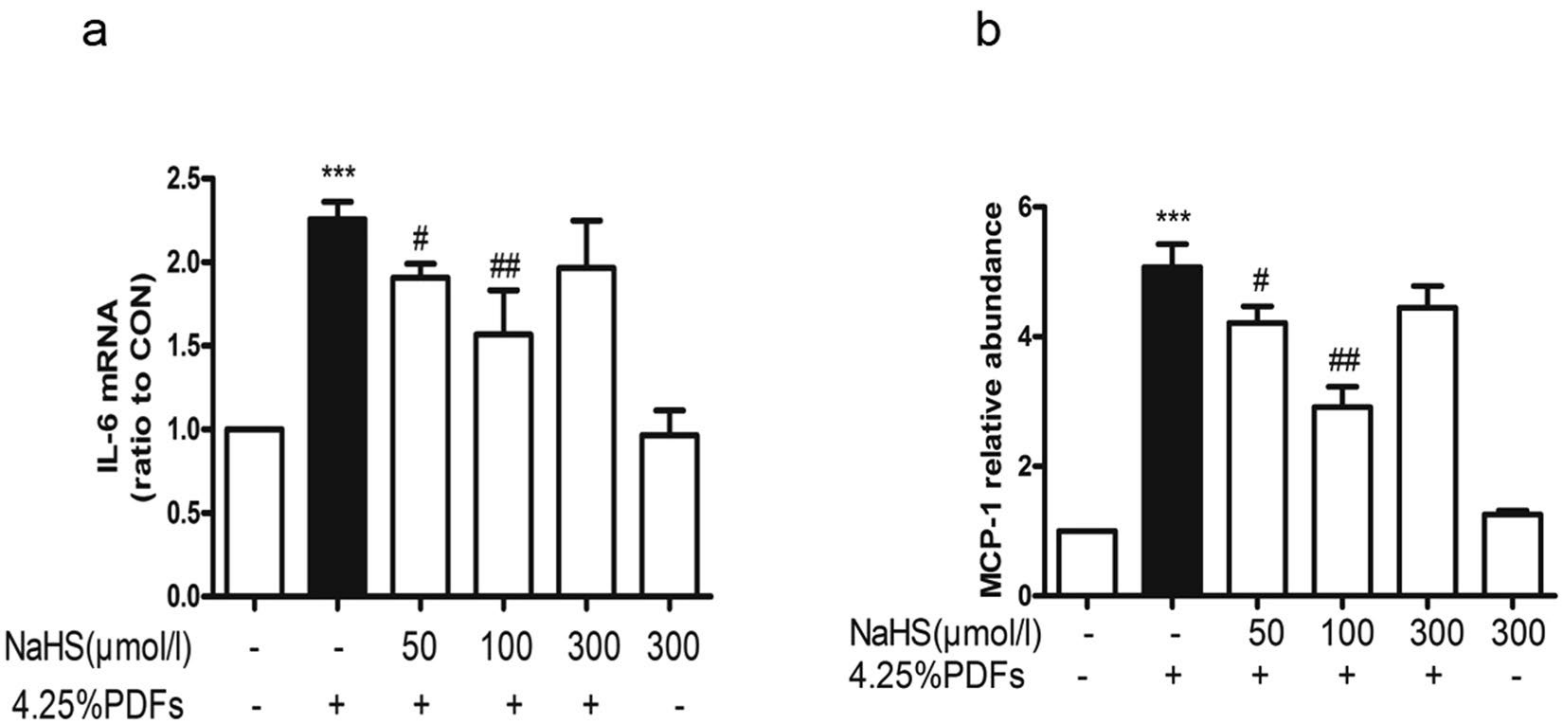
Effect of H_2_S on inflammatory factors induced by 4.25% glucose PDFs in RPMCs. RPMCs were pretreated with or without NaHS(50, 100, 300 μmol/L) for 30 min and then exposed to the mixture of 4.25% glucose PDFs and culture medium by 1:1 for 6 h. (**a**,**b**) IL-6 and MCP-1 relative abundance of mRNA were normalized by β-actin RNA expression. Data represent mean ± SD of three independent experiments. ***P < 0.001 versus control group; ^##^*P* < 0.01, ^#^*P* < 0.05 versus 4.25% glucose PDFs group.

RPMC were treated with various concentrations of NaHS (50–300 μmol/L) for 30 mins, then exposed to the mixture of 4.25% glucose PDFs and culture medium by 1:1 for 1 h. 4.25% glucose PDFs stimulated the phosphorylation of Smad 3 and Smad 2/3, which were decreased by the addition of 50–300 μmol/L NaHS (Fig. 5).

### H_2_S reduces the inflammatory cytokines activated by 4.25%PDFs in RPMC

**Figure 6:**
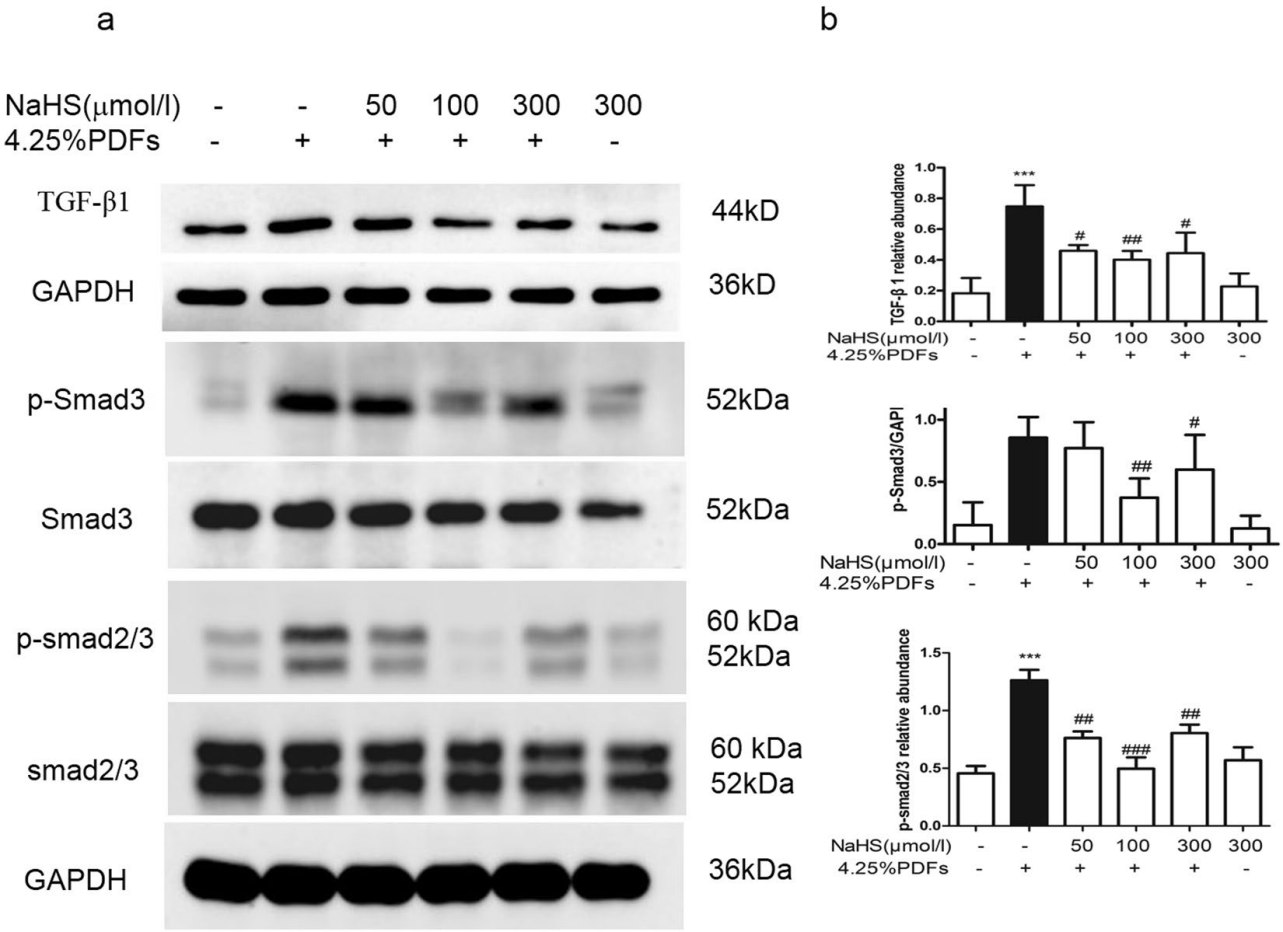
Effect of H_2_S on TGF-β1 signal pathway activated by 4.25% glucose PDFs in RPMCs. RPMCs were incubated with or without NaHS (50, 100, 300 μmol/L) for 30 min, followed by the stimulation of the mixture of 4.25% glucose PDFs and culture medium by 1:1 for 1 h. (**a**) Representative images of TGF-β1, phospho-Smad3, phospho-Smad2/3 are presented and (**b**) relative abundance of these proteins in each group are calculated. Data represent mean ± SD of three independent experiments. **P < 0.01 versus control group; ^#^P < 0.05, ^##^P < 0.01 versus 4.25% glucose PDFs group.

RPMCs were pretreated with or without NaHS (50, 100, 300 μmol/L) for 30 min. Cells were then treated with 4.25% glucose PDFs in culture medium (1:1) for 6 h. The cellular inflammatory cytokines including IL-6 and monocyte chemoattractant protein-1(MCP-1) were determined by real-time PCR. IL-6 and MCP-1 were increased by the stimulation with 4.25% glucose PDFs, but were decreased after pre-treatment with NaHS. The optimal effect of NaHS on cytokines was observed at 100 μmol/L (Fig. 6).

## Discussion

The present study demonstrated that high glucose peritoneal solution induced epithelial-mesenchymal transition (EMT) in primarily cultured peritoneal mesothelial cells. Administration with exogenous H_2_S is able to reverse EMT in peritoneal mesothelial cells by inhibiting the TGF-β1-smad3 signal pathway and the production of inflammatory cytokines including IL-6 and MCP-1. To the best of our knowledge, this is the first work to confirm the inhibitory effect of EMT of H_2_S in peritoneal mesothelial cells.

Although a recent study using genetic labeling technique indicated that submesothelial fibroblasts were the major source of myofibroblast in peritoneal fibrosis^7^, the potential role of EMT in peritoneal fibrosis could not be totally excluded. Animal and clinical studies have confirmed *in situ* evidence of EMT and interventions aiming at EMT also improved peritoneal fibrosis^8–10^. Consistent with a previous study^11^, our data showed that high glucose peritoneal dialysis fluid stimulated the mesothelial cells to undergo EMT characterized by the loss of epithelial tight junction molecules such as ZO-1 and cytokeratin as well as the increase of myofibroblast marker of α-SMA. Exogenous NaHS reversed such a phenotype shift in the mesothelial cells. NaHS also decreased the release of profibrotic factor including PAI-1 and TGF-β1 and reduced the production of extracellular matrix protein including fibronectin. Although 300 μmol/L NaHS was not toxic to the mesothelial cells and effective in alleviating the EMT process in our study, the most effective dose of NaHS against the differentiation of mesothelial cells to myofibrobalst was 100 μmol/L, which has been thought to produce a physiologically concentration of H_2_S in many previous studies^12,13^. Such a finding is also consistent with our previous work that relatively small dose of NaHS is preferable in the treatment of renal fibrosis in UUO animal model^14^.

Peritoneal inflammation plays an important role in mesothelial cell EMT^15^. It is believed that EMT is a pathological event responding to trauma and inflammatory insult. Numerous studies have confirmed that H_2_S is able to inhibit inflammation in multiple organs^16^. We previous found that lower dose of NaHS (5.6–56 μg/kg.d) was able to inhibit inflammation of UUO animal model by suppressing the MAPK signal pathway, while higher dose of NaHS (560 μg/kg.d) aggravated inflammation^14^. The current study demonstrated that NaHS 100 μmol/L was effective to reduce cellular inflammatory cytokines of IL-6 and MCP-1 induced by 4.25% glucose PDFs, indicating that inhibitory effect of H_2_S on EMT may contributable to the anti-inflammation property of H_2_S.

TGF-β1 was firstly proven to induce EMT in mammary epithelial cells^17^. Even since then, a number of epithelial cell types such as renal tubular cells and alveolar epithelial cells have been shown to undergo EMT stimulated by TGF-β1^18,19^. TGF-β1 can induce EMT through Smad dependent and independent signal pathway. In the Smad dependent way, TGF-β1 induces the phosphorylation of Smad proteins including Smad 2 and Smad3 that control the transcription of many fibrogenic genes of EMT including α-SMA, fibronectin, PAI-1 and MCP-1^20^. Our results demonstrated that high glucose PDFs stimulated the production of TGF-β1 as well as the phosphorylation of Smad3 and Smad2/3, while exogenous H_2_S partially inhibited such an effect. These data support that the anti-EMT effect of H_2_S in peritoneal mesothelial cells is associated with the inhibition of TGF-β1-Smad signal pathway.

In conclusion, our data suggested that 4.25% glucose PDFs stimulated EMT of peritoneal mesothelial cells characterized by the loss of cell-cell adhesion protein including ZO-1 and the increase of parenchymal marker of α-SMA. Exogenous H_2_S inhibited the EMT process induced by high glucose PDFs due to its anti-inflammation property as well as the inhibition of the TGF-β1-Smad signal pathway.

## Materials and Methods

### Animal

Eight-week-old male Sprague–Dawley (SD) rats weighing 200–240 g were purchased from Soochow University Laboratory Animal Center, and raised in an environment at 24 ± 2 °C with a 12 hours light/dark cycle. All experimental protocols were approved by the Ethics Committee of the Second Affiliated Hospital of Soochow University. Animal experiment conforms to the international guidelines of use and care of laboratory animals.

### Dialysis animal model study

To achieve peritoneal dialysis rats, 20 ml of 4.25% -glucose PD fluid (Baxter International Inc., Chicago, Ill., USA) was administered to SD rats daily for 28 days, with 0.6 mg/kg of LPS (Sigma, St. Louis, Mo., USA) in the PD fluid on days 1, 3, 5, and 7. Rats were randomly divided into four groups: (1) control group (20 ml saline); (2) PD group; (3) PD + NaHS (Sigma, St. Louis, Mo., USA) group: (56 µg/kg/day NaHS daily in PD fluid with LPS); (4) NaHS group (56 µg/kg/day NaHS daily in 20 ml saline). All drugs were given intraperitoneally. On the 28th day, rats were sacrificed and the peritonea of the upper right abdominal wall were collected.

### Histological analysis

Peritoneal tissue was fixed in 4% paraformaldehyde solution and embedded in paraffin. Sections were made at 4 μm thick, deparaffined and stained using Masson reagents according to the manufacturer’s manual (Solarbio, Perking, China). Sections were then observed under a light microscopy and the amount of collage (blue area) was quantified in five selected fields (200×).

### Cell culture study

Rat peritoneal mesothelial cells (RPMCs) were isolated and cultured as previously described^21^. Concisely, rat omentum were obtained from male SD rats weighing 130–140 g and digested with 0.125% trypsin- ethylenediamine tetracetic acid (EDTA) for 15 mins in incubator shakers (BiuBard,150 rpm, 7 °C). RPMCs were then centrifuged at 1000 rpm for 5 mins and the cell pellets were suspended. The cells were then cultured in DMEM/F12 medium supplemented with 15% fetal bovine serum (FBS), 100 U/mL penicillin, 100 μg/mL Streptomycin (Invitrogen, Carlsbad, CA) and 0.5 ug/ml transferring (Sigma). Cells of the third passage were used for the experiments.

### Immunofluorescence Staining

In order to identify RPMCs, monolayer cells were cultured on glass coverslips in complete culture medium to 70% confluence. Cells were fixed with 4% paraformaldehyde for 25 mins and permeabilized with 0.2% Triton X-100 for 5 mins. The coverslips were incubated with mouse anti-vimentin antibody or mouse anti-cytokeratin antibody (1:100 dilution; Santa Cruz Biotechnology, Inc., USA) overnight at 4 °C. Coverslips were then incubated with second antibody (1:2,000 dilution; KPL, USA) for 1 h at room temperature and counter-stained with DAPI (1:500).Then coverslips were mounted in 80% glycerol in PBS, finally photographed with a Nikon fluorescence photomicroscope.

### Quantitative PCR

The expression of RNA was evaluated by real-time RT-PCR in RPMCs. Total RNA was isolated from RPMC by the Trizol reagent (Life technologies, USA) according to the manufacturer’s instruction. After reverse transcription, RT-PCR amplification was performed using the SYBR Green Master Mix (Thermo Fisher Scientific). The RT-PCR amplification procedure was constitutive of denaturation at 94 °C for 30 s, annealing at 50 °C for 30 s, and extension at 72 °C for 1 min after a 3 min denaturation step at 94 °C in a Applied Biosystem PCR System 9700 (Bio-RAD PTC-200,USA). The primer sequences of β-actin, MCP-1 and IL-6 were synthesized by (GenePharma, Shanghai, China). Primer sequences are: forward 5′-GTGCTATGTTGCTCTAGACTTCG-3′, reverse 5′ATGCCACAGGATTCCATACC-3′ (β-actin); forward 5′-CCCCACTGATACGCCTGAG-3′, reverse 5′-GGACTGATCCCATTGATTTCC-3′ (IL-6); forward 5′- GCAGGTGTCCCAAAGAAG-3′, reverse 5′- TCAAAGGTGCTGAAGTCC-3′ (MCP-1). Relative abundance of mRNA was normalized by β-actin RNA expression.

### Western Blotting

A total of 20 μg protein was loaded and separated on 10% SDS-PAGE. After electrophoresis, proteins were transferred to a PVDF membrane and blocked with 5% milk/Tris-buffered saline and Tween-20 buffer. The membranes were incubated with ZO-1 (1:1000), cytokeratin (1:1000), plasminogen activator inhibitor 1(PAI-1) (1:1000), fibronectin (1:1000), TGF-β1 (1:1000), phosphor-Smad3 (1:500) and phosphor-Smad2/3 (1:500) at 4 °C overnight. Afterwards, the membranes were washed and incubated at room temperature for 1 h with the secondary antibody and developed with an ECL kit (Biological Industries, China).The image was captured with the GeneGenius imaging system (Syngene, Cambridge, UK). Band intensity was measured with the Image J software (Bethesda, MD). All primary and second antibodies were purchased from Santa Cruz Biotechnology (Santa Cruz, CA).

### Statistical analysis

Data are represented as mean ± S.D of three independent experiments. One-way ANOVA analysis was used to determine the variance among multiple groups. P < 0.05 was defined statistically significance.
